# The Serum Melatonin Levels and Mortality of Patients with Spontaneous Intracerebral Hemorrhage

**DOI:** 10.3390/brainsci9100263

**Published:** 2019-10-01

**Authors:** Leonardo Lorente, María M. Martín, Pedro Abreu-González, Luis Ramos, Mónica Argueso, Jordi Solé-Violán, Juan J. Cáceres, Alejandro Jiménez, Victor García-Marín

**Affiliations:** 1 Intensive Care Unit, Hospital Universitario de Canarias, Ofra s/n, La Laguna, 38320 Santa Cruz de Tenerife, Spain; 2 Intensive Care Unit, Hospital Universitario Nuestra Señora de Candelaria, Crta del Rosario s/n, 38010 Santa Cruz de Tenerife, Spain; mar.martinvelasco@gmail.com; 3 Deparment of Phisiology, Faculty of Medicine, University of the La Laguna, 38320 La Laguna, Santa Cruz de Tenerife, Spain; pabreu@ull.es; 4 Intensive Care Unit, Hospital General La Palma, Buenavista de Arriba s/n, Breña Alta, 38713 La Palma, Spain; lramosgomez@gmail.com; 5 Intensive Care Unit, Hospital Clínico Universitario de Valencia, Avda. Blasco Ibáñez nº17-19, 46004 Valencia, Spain; moni_begasa@hotmail.com; 6 Intensive Care Unit, Hospital Universitario Dr. Negrín, CIBERES, Barranco de la Ballena s/n, 35010 Las Palmas de Gran Canaria, Spain; jsolvio@gobiernodecanarias.org; 7 Intensive Care Unit, Hospital Insular, Plaza Dr. Pasteur s/n, 35016 Las Palmas de Gran Canaria, Spain; juanjose.caceresagra@gobiernodecanarias.org; 8 Research Unit, Hospital Universitario de Canarias, Ofra s/n. La Laguna, 38320 Santa Cruz de Tenerife, Spain; ajimenezsosa@gmail.com; 9 Department of Neurosurgery, Hospital Universitario de Canarias, Ofra, s/n. La Laguna, 38320 Santa Cruz de Tenerife, Spain; vicgarmar666@gmail.com

**Keywords:** spontaneous intracerebral hemorrhage, melatonin, patients, mortality

## Abstract

**Objective**: Providing melatonin in animal models with spontaneous intracerebral hemorrhage (SIH) has been associated with beneficial effects. However, to our knowledge, there are no published data on circulating melatonin levels regarding the prognosis of SIH patients. Therefore, the objectives of this study were to determine whether serum melatonin levels in SIH patients were associated with early mortality and whether they could be used as prognostic biomarkers. **Methods**: This observational and prospective study included patients with supratentorial and clinically severe SIH (defined as Glasgow Coma Scale GCS <9) admitted to the Intensive Care Units of six Spanish hospitals. Serum melatonin levels were determined at the time of severe SIH diagnosis. Mortality at 30 days was the study end-point. **Results**: Non-surviving patients (*n* = 46) showed higher serum melatonin levels (p < 0.001) than surviving (*n* = 54) patients. An area under the curve was found for the prediction of 30-day mortality by serum melatonin levels of 0.89 (95% CI = 0.81–0.94; p < 0.001). Multiple logistic regression analysis showed an association of serum melatonin levels with 30-day mortality (Odds Ratio = 8.16; 95% CI = 2.30–28.95; *p* = 0.001) after controlling for midline shift, glycemia, early evacuation of SIH, and Intracerebral hemorrhage (ICH) score. **Conclusions**: The novel findings by our study were the presence of higher serum melatonin levels in non-surviving patients than in surviving patients and the association of these levels with mortality.

## 1. Introduction

A large number of deaths and disabilities as well as the need for a high consumption of care resources are caused by spontaneous intracerebral hemorrhage (SIH) [1]. Oxidative stress has been implicated in the secondary brain injury of SIH [2,3,4]. 

Melatonin, a lipophilic amino acid derived from tryptophan, is synthesized in the pineal gland and also on other organs such as the retina, thymus, bone marrow, and gastrointestinal tract [5,6]. Melatonin has a role in sleep regulation, shows antioxidant effects due to upregulation of some antioxidant enzymes, and is a potent reactive oxygen species scavenger [7,8]. The administration of melatonin in animal models with SIH has been associated with beneficial effects, such as the reduction of oxidative damage and motor dysfunction [9,10]. Previously, we found a higher oxidative state in non-survivor SIH patients than in survivor patients, as assessed by serum concentrations of malondialdehyde [11] (biomarker of lipid peroxidation) [12,13]. However, to our knowledge, there are no published data about circulating melatonin levels regarding the prognosis of SIH patients. Therefore, the objectives of this study were to determine whether serum melatonin levels in SIH patients were associated with early mortality and whether they could be used as prognostic biomarkers. 

## 2. Methods 

### 2.1. Design and Subjects

This observational and prospective study was made with the approval by the Review Board of the six Spanish hospitals participating: H. Clínico Universitario de Valencia, Universitario Nuestra Señora de Candelaria (Santa Cruz de Tenerife), H. General de La Palma, H. Universitario Dr. Negrín (Las Palmas de Gran Canaria), H. Universitario de Canarias (San Cristóbal de La Laguna), and H. Insular de Las Palmas de Gran Canaria. Written and signed informed consent was obtained by a family member of each patient.

Patients with supratentorial SIH and clinically severe (defined as Glasgow Coma Scale (GCS) [14] <9) admitted to Intensive Care Units were included in the study. Patients with infratentorial hemorrhage, traumatic hemorrhage, hemorrhagic transformation of brain infarction, age <18 years, pregnancy, inflammatory disease, or malignant disease were excluded from the study.

### 2.2. Variables Recorded

Epidemiological variables such as age and sex were collected. Blood variables were also collected such as the international normalized ratio (INR), activated partial thromboplastin time (aPTT), platelets, fibrinogen, sodium, lactic acid, creatinine, glycemia, and the arterial oxygen pressure/fraction of inspired oxygen ratio (PaO_2_/FIO_2_). The following scores were recorded: the Acute Physiology and Chronic Health Evaluation II (APACHE II) score [15], GCS, and the intracerebral hemorrhage (ICH) score [16]. Additionally, the site, volume, and cause of SIH, as well as the presence of intraventricular hemorrhage, hydrocephalus, transtentorial herniation, or midline shift were also recorded. In addition, we registered early SIH evacuation (within first 24 hours of SIH diagnosis), and mortality at 30 days (which was considered the study end-point).

### 2.3. Blood Samples and Determination of Serum Melatonin Levels 

Serum samples at moment of severe SIH diagnosis were taken and frozen at −80 °C until its analysis in the Physiology Department at Faculty of Medicine of University of La Laguna (Tenerife, Spain). Serum melatonin determinations were made using a kit from Immuno Biological Laboratories (IBL Hamburg GmbH, Hamburg, Germany). The assay had a detection limit of 0.13 pg/mL, an intra-assay variation coefficient of 6.4%, and an inter-assay variation coefficient of 11.1%. 

### 2.4. Statistical Methods

Frequencies and percentages were used to report categorical variables, and medians and interquartile ranges were used to report continuous variables. The Chi-square test was used to compare categorical variables between surviving and non-surviving patients, and the Wilcoxon-Mann-Whitney test was used to compare continuous variables. The prediction capacity of mortality at 30 days involving serum melatonin levels was estimated by performing a receiver operating characteristic (ROC) analysis. To determine the association of serum melatonin levels with 30 day-mortality, a multiple logistic regression analysis was performed controlling for midline shift, glycemia, and early evacuation of SIH and ICH scores; while an odds ratio and its 95% confidence intervals (CI) were used to estimate the clinical impact of the variables included in the analysis. Kaplan-Meier curves were constructed using serum melatonin levels > 3.94 pg/mL (because it was the optimal cut-off according to the Youden J index) and mortality at 30 days. To do the statistical analyses, the programs NCSS 2000 (Kaysville, UT, USA), LogXact 4.1 (Cytel Co., Cambridge, MA), and SPSS 17.0 (SPSS Inc., Chicago, IL, USA) were used. *p*-values < 0.05 were considered statistically significant.

## 3. Results

Non-surviving (*n* = 46) patients showed higher glycemia (*p* = 0.01), older age (*p* = 0.006), ICH score (*p* < 0.001), APACHE-II score (*p* < 0.001), midline shift (*p* = 0.005) and intracerebral hemorrhage volume (*p* = 0.02), and lower GCS (*p* < 0.001) than surviving SIH patients (*n* = 54). In addition, non-surviving patients had higher serum melatonin levels than surviving patients (*p* < 0.001) (Table 1). 

**Figure 1:**
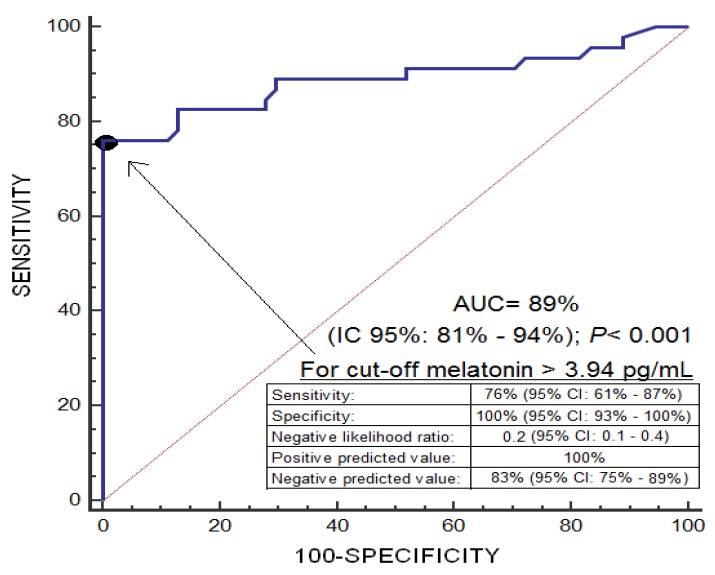
Receiver operation characteristic analysis using serum melatonin levels as a predictor of mortality at 30 days.

**Figure 2:**
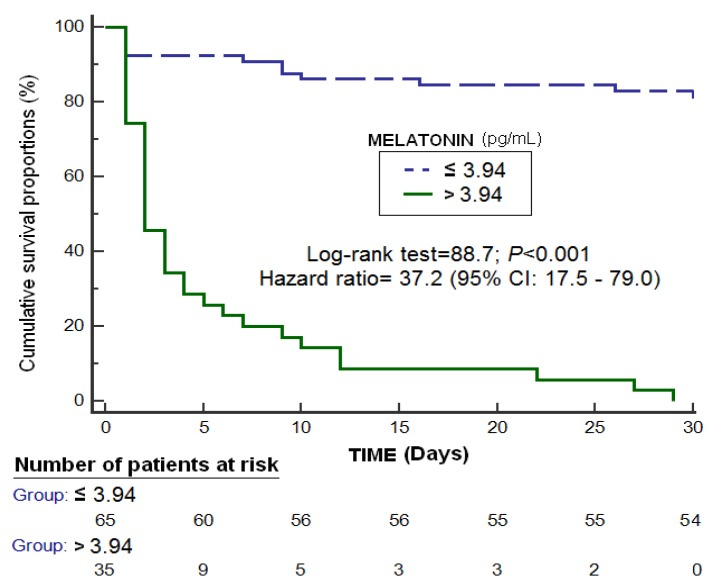
Survival curves at 30 days using serum melatonin levels >3.94 pg/mL as the cut-off.

An area under the curve was found for the prediction of 30-day mortality by serum melatonin levels of 0.89 (95% CI = 0.81–0.94; *p* < 0.001) (Figure 1). Kaplan-Meier analysis showed higher mortality at 30 days in patients with serum melatonin levels > 3.94 pg/mL (Hazard ratio = 37.2; 95% CI = 17.5–79.0; *p* < 0.001) (Figure 2). Multiple logistic regression analysis showed an association of serum melatonin levels with 30-day mortality after controlling for midline shift, glycemia, early evacuation of SIH, and the ICH score (Odds Ratio = 8.16; 95% CI = 2.30–28.95; *p* = 0.001), and after controlling for age, GCS, volume of SIH and intraventricular hemorrhage (Odds Ratio = 7.75; 95% CI = 2.05–29.27; *p* = 0.003) (Table 2). A positive association between serum levels of melatonin and malondialdehyde was found (rho = 0.43; *p* < 0.001); but not between serum melatonin levels with age (rho = 0.19; *p* = 0.06) and the SIH volume (rho = 0.11; *p* = 0.31).

## 4. Discussion

To our knowledge, our study showed serum melatonin levels in SIH patients for first time. The novel findings of our study were the presence of higher serum melatonin levels in non-surviving than in surviving patients and the association of those levels with lipid oxidation and mortality. Two regression models with five variables were constructed to avoid an over-fitting effect because 46 non-survivor patients were recorded in our study. The variables included in the first model were serum melatonin levels, glycemia, early SIH evacuation, ICH score, and midline shift, and the variables associated with mortality were the serum melatonin levels and midline shift. The variables included in the first model were age, volume of SIH, GCS, and intraventricular haemorrhage because those variables are included in the ICH score. This model also included serum melatonin levels, and the variables associated with mortality were serum melatonin levels, age and GCS. Our findings are consistent with the findings from previous studies showing an association between high serum melatonin concentrations and mortality in patients with traumatic brain injury [17] or brain infarction [18].

Previously, serum malondialdehyde were determined in these patients and higher levels were found in non-surviving patients than in surviving patients [11]. In our current study, a positive association was found between circulating levels of melatonin and malondialdehyde. *Malondialdehyde* is a lipid peroxidation product that appears due to oxidation of phospholipids from cell membranes, which is released to extracellular space and finally appears in blood [12,13]. In addition, this association between high circulating malondialdehyde levels and mortality has been found in patients with traumatic brain injury [19] or cerebral infarction [20].

Another interesting point is that the administration of melatonin in SIH animal models have reduced oxidative damage [21,22,23,24,25] and motor deficit [21]. We believe that higher serum levels of melatonin and malondialdehyde in non-surviving patients compared to surviving SIH patients, and the association between serum levels of melatonin and malondialdehyde could mean that the non-survivors have higher oxidants species production, which is the origin of a higher oxidative damage (with higher serum malondialdehyde levels) and higher serum melatonin levels (attempting to maintain a balance between antioxidant and oxidant states). However, those higher serum melatonin levels in non-surviving patients are not able to compensate for the high production of oxidant species, and they contribute to brain damage and ultimately to patient death.

We must recognize some limitations of our study, such as the fact that we have not assessed circulating melatonin levels during the evolution of the patient and other oxidative damage products. In addition, we have not determined blood melatonin levels in healthy subjects; although the levels found in other series were 15.15 ± 1.65 pg/mL [26]. However, we believe that our findings in SIH patients could motivate research on melatonin in SIH patients.

## 5. Conclusions

The novel findings of our study were the presence of higher serum melatonin levels in non-surviving patients than in surviving patients and the association of these levels with mortality.

## Figures and Tables

**Table 1:** Clinical and biochemical characteristics of 30-day surviving and non-surviving patients with spontaneous intracerebral hemorrhage (SIH).

	Non-Surviving (*n* = 46)	Surviving (*n* = 54)	*p*-Value
Gender female—*n* (%)	17 (37.0)	17 (31.5)	0.67
Age (years)—median (p 25–75)	68 (57–74)	59 (52–67)	0.006
Site of SIH—*n* (%)			0.81
Lobar	33 (71.7)	41 (75.9)	
Basal ganglia	4 (8.7)	3 (5.6)	
Thalamus	3 (6.5)	5 (9.3)	
Periventricular	6 (13.0)	5 (9.3)	
Volume of SIH (cc)—median (p 25–75)	68 (29–99)	38 (17–62)	0.02
Transtentorial herniation—*n* (%)	1 (2.2)	2 (3.7)	0.99
Midline shift (mm)—median (p 25–75)	5 (0–11)	1 (0–7)	0.005
Hydrocephalus—*n* (%)	26 (56.5)	21 (38.9)	0.11
Intraventricular hemorrhage—*n* (%)	23 (50.0)	17 (31.5)	0.07
Cause of SIH—*n* (%)			0.07
Hypertension	30 (65.2)	37 (68.5)	
Amyloid angiopathy	4 (8.7)	2 (3.7)	
Aneurysm	0	3 (5.6)	
Arteriovenous malformation	0	5 (9.3)	
OAT in therapeutic range	6 (13.0)	3 (5.6)	
OAT out of therapeutic range	6 (13.0)	3 (5.6)	
Fibrinolytic treatment	0	1 (1.9)	
ICH score—median (p 25–75)	3 (2–3)	2 (1–2)	<0.001
GCS—median (p 25–75)	4 (3–6)	8 (6–8)	<0.001
APACHE-II score—median (p 25–75)	24 (20–26)	18 (14–20)	<0.001
PaO2/FI0_2_ ratio—median (p 25–75)	289 (215–397)	270 (189–350)	0.40
Creatinine (mg/dL)—median (p 25–75)	0.80 (0.60–1.01)	0.77 (0.68–0.90)	0.36
Sodium (mEq/L)—median (p 25–75)	139 (135–143)	139 (137–142)	0.93
Glycemia (g/dL)—median (p 25–75)	170 (141–216)	141 (118–190)	0.01
Lactic acid (mmol/L)—median (p 25–75)	1.80 (1.30–2.55)	1.70 (1.00–2.51)	0.23
INR—median (p 25–75)	1.14 (1.02–1.87)	1.10 (1.00–1.31)	0.34
Fibrinogen (mg/dL)—median (p 25–75)	382 (350–510)	390 (280–493)	0.34
Platelets—median × 10^3^/mm^3^ (p 25–75)	198 (159–270)	193 (145–252)	0.57
aPTT (s)—median (p 25–75)	30 (24–34)	29 (27–32)	0.68
Early evacuation of SIH—*n* (%)	9 (19.6)	18 (33.3)	0.18
Melatonin (pg/mL)—median (p 25–75)	4.78 (3.90–11.10)	2.62 (2.17–3.08)	<0.001

*p* 25–75 = 25th–75th percentile; OAT = Oral anticoagulant treatment; ICH = intracerebral hemorrhage; GCS = Glasgow Coma Scale; APACHE II = Acute Physiology and Chronic Health Evaluation; PaO_2_ = arterial oxygen pressure; FIO_2_ = fraction inspired oxygen; INR = international normalized ratio; aPTT = activated partial thromboplastin time.

**Table 2:** Logistic regression analysis to predict 30-day mortality.

Variable	Odds Ratio	95% Confidence Interval	*p*
**First model**			
Serum melatonin levels (pg/mL)	8.16	2.30–28.59	0.001
Glycemia (g/dL)	0.99	0.97–1.01	0.23
Early evacuation of SIH (yes vs. non)	0.11	0.01–1.07	0.06
ICH score (points)	2.86	0.87–9.38	0.08
Midline shift (mm)	1.27	1.05–1.53	0.01
**Second model**			
Serum melatonin levels (pg/mL)	7.75	2.05–29.27	0.003
Age (years)	1.11	1.01–1.22	0.03
Volume of SIH (cc)	1.02	0.99–1.04	0.08
GCS (points)	0.37	0.21–0.66	0.001
Intraventricular hemorrhage (yes vs. non)	1.26	0.24–6.67	0.79

SIH = spontaneous intracerebral hemorrhage; ICH = intracerebral hemorrhage.
